# Supplementary figures and images for: Meat Substitute Development from Fungal Protein (Aspergillus oryzae)
Source: Foods. 2022 Sep 20;11(19):2940. doi: 10.3390/foods11192940 (PMC9563988; doi:10.3390/foods11192940)

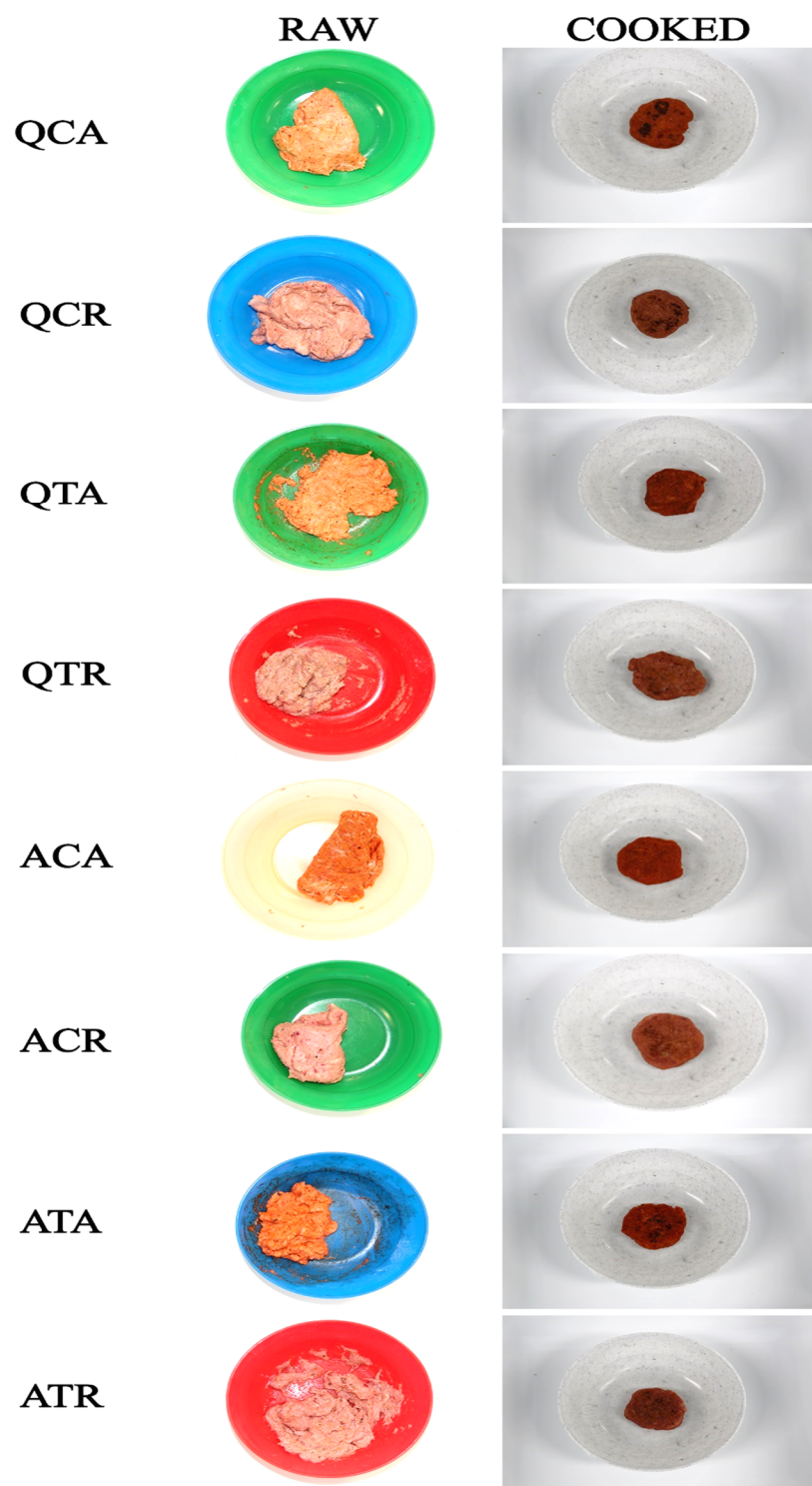

**Figure S1** The appearance of the raw and cooked formulation

Supplement: Supplementary file 1 [file foods-11-02940-s001.zip › Figure S1.pdf]

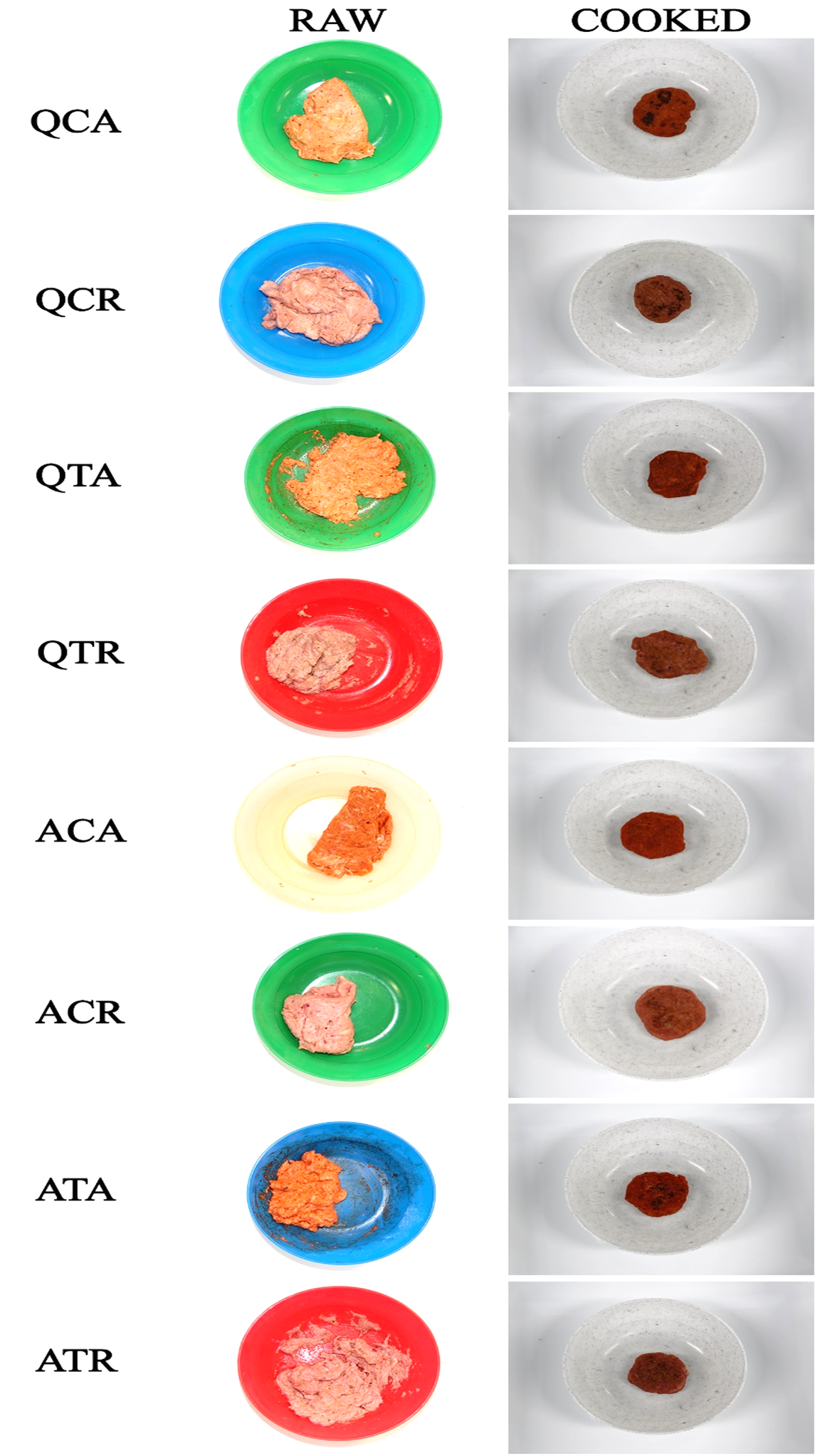

Supplement: Supplementary file 1 [file foods-11-02940-s001.zip › Figure S1.png]
